# Exploration for adequate non-diffractive beam generation in dense scattering media

**DOI:** 10.1038/s41598-022-12810-4

**Published:** 2022-05-25

**Authors:** Alifu Xiafukaiti, Nofel Lagrosas, Tatsuo Shiina

**Affiliations:** Graduate School of Engineering, Chiba University, 1-33 Yayoi-cho, Inage-ku, Chiba 263-8522 Japan

**Keywords:** Optics and photonics, Physics

## Abstract

The propagation methods of a non-diffractive beam (NDB) for optical sensing in scattering media have been extensively studied. However, those methods can realize the high resolution and long depth of focus in the viewpoint of microscopic imaging. In this study, we focus on macroscopic sensing in living tissues with a depth of a few tens centimeters. An experimental approach for generating adequate NDB in dense scattering media based on the linear relationship between propagation distance and transport mean free path is reported. For annular beams with different diameters, the same changes of the center intensity ratio of NDB are obtained from the experiment results. They are discussed with theoretical analysis. As a result, the maximum center intensity ratio of the adequate generated NDB can be estimated at arbitrary propagation distance in the dense scattering media.

## Introduction

Optical sensing methods have been widely developed for biomedical imaging^1–3^ and environmental measurements^4–6^. As an example, optical coherence tomography (OCT) is a non-invasive ophthalmologic diagnostic technique that renders an in-vivo cross-sectional view of the dense scattering media such as biomedical tissues up to a depth of less than 2 mm^7–9^. On the other hand, for long-range sensing, lidar is widely used for measuring optical properties of atmospheric particles and molecules that have smaller concentrations than biomedical tissues^10^.

Laser beams with annular-shaped intensity distributions have attracted much attention for a wide variety of applications such as particle trapping^11^, laser manipulation of biological cells^12^, laser writing^13^, microscopy^14^ and lidar system^15^. In general, the propagation stability of light is decreased due to effects of diffraction and scattering^16^. Using the self-transformation property of an annular beam into a non-diffractive beam (NDB) is a method for increasing beam propagation stability^17–19^. NDB, also known as quasi-Bessel beam, retains its intensity distribution over a limited spatial range while the intensity changes over the axial range of the beam propagation^20,21^. Several studies of NDB have shown that the main lobe of the NDB is more stable during the propagation through a scattering and absorbing media^22–26^. Since the width of the main lobe is narrower than the diffraction limit, this NDB can be utilized in high-resolution optical sensing. On the other hand, NDBs have a function to self-reconstruct when it encounters an obstacle^27,28^. For this merit, NDB will offer a good alternative solution for optical sensing in scattering media. In those recent studies, the sensing detection limit of scattering media is up to a few millimeters in the viewpoint of microscopic imaging even though NDB is applied. To establish macroscopic sensing of scattering media such as living tissues, we focused on the propagation property of the annular beam in dense scattering media in our previous work^29–32^. The NDB was generated under the propagation of a few tens centimeters distance in the dense scattering media with a scattering coefficient of a few cm^−1^, which is near to that of human tissues^33^.

To realize the macroscopic sensing of scattering media, the condition for generating NDB based on the appropriate combination among propagation distance, media concentration and beam size is explored in this work. Therefore, we have the following objectives: (1) to explore the trend of the self-transformation property of annular beams with different diameters to generate NDB in scattering media; (2) to estimate the conditions for the adequate NDB generation using the linear relationship between the propagation distance and the transport mean free path (TMFP) in scattering media; and (3) to establish the relationship between center intensity ratio of NDB and propagation distance in scattering media by experimental and theoretical approaches. Here, we define the adequate NDB as the maximum central intensity under a certain propagation distance in scattering media. The former publication reported the knowledge of NDB generation in scattering media. This work reports a new finding on how to generate adequate NDBs under varying beam and media conditions. This will deduce important information on the common characteristics underlying the generation of NDB.

## Methods and results

**Figure 1:**
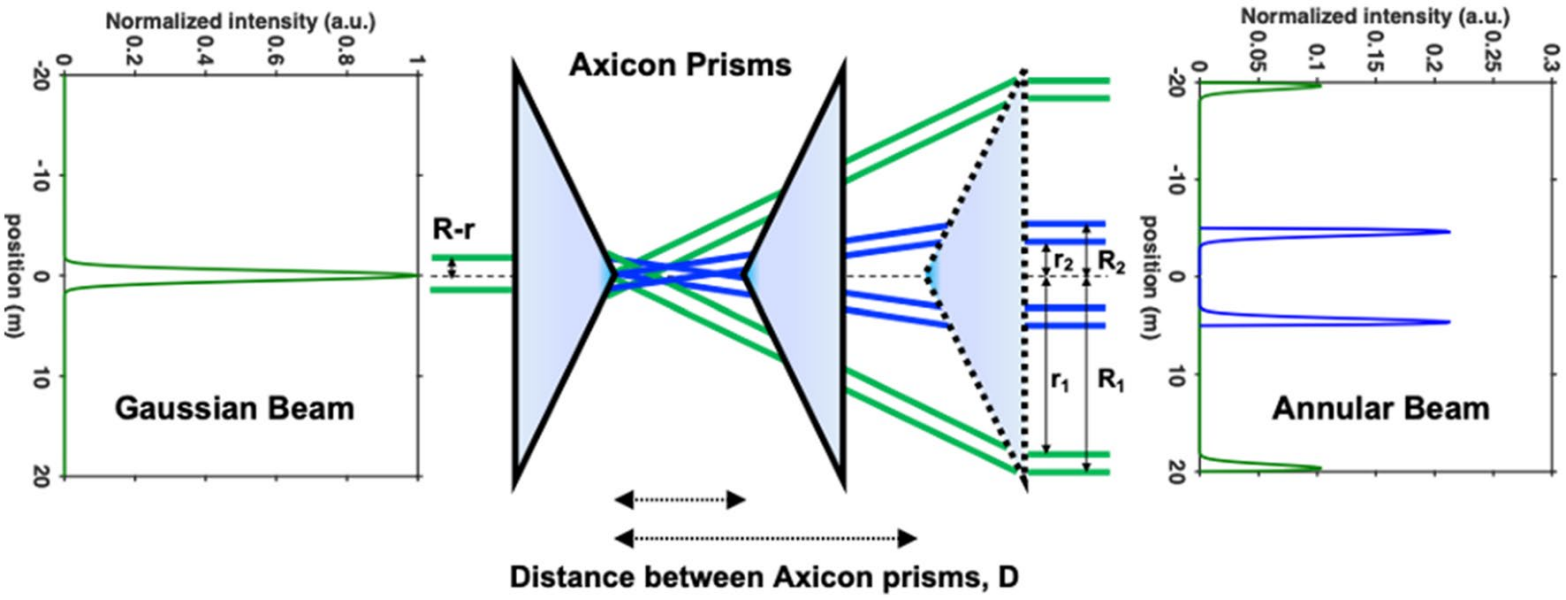
Transformation from the Gaussian beam to the annular beam with different diameters using a pair of Axicon prisms. The left and right-side graphs show the intensity distributions of Gaussian beam and annular beams with diameters 10 mm (blue) and 40 mm (green), respectively.

Generation of the annular beam can be realized by various approaches such as Fresnel zone plate, Axicon and thin films^34–36^. However, for producing a collimated annular beam, illumination of a Gaussian beam to a pair of Axicon prisms is an efficient method that can easily adjust the intensity and width of the annular beam^37^. When a collimated Gaussian beam is incident on the first Axicon prism, a Bessel-gauss beam^38,39^ is formed, and this beam passes through the second Axicon prism to produce a collimated annular beam. A pair of Axicons with the same apex angle are arranged so that the two tips are in the same optical axis as the incident beam. Also, the large apex angle of the Axicon ensures that the beam has the same polarization property as the incident beam. Depend on the pair of Axicon prisms properties, the diameter *d*_A_ of an annular beam is expressed as

1 $$ d_{{\text{A}}} \left( r \right) = 2{\text{D}} \cdot \tan \left[ {\upbeta \left( {{\text{n}} - 1} \right)} \right] , $$

where D, n and β are the distance between the axicon prisms, the refractive index, and apex angle of the Axicon prism, respectively. The diameter of the annular beam can be changed by adjusting the distance D between the two prisms as shown in Fig. 1. The amplitude distribution of a Gaussian beam *u*_G_(*r*) is expressed as

2 $$ u_{{\text{G}}} \left( r \right) = \frac{1}{{\uppi \omega^{2} }}\exp \left( { \frac{{ - r^{2} }}{{\omega^{2} }}} \right) , $$

where *ω* is the distance from the center to the position where the amplitude of the Gaussian distribution becomes e^−1^ of the center value, and *r* represents the radial point of the plane perpendicular to the propagation direction of light. Through the Axicon prisms, the annular ring and the Gaussian beam are related using Eq. 3,

3 $$ u_{{\text{G}}} \left( {R - r} \right)^{2} \cdot 2\uppi \left( {R - r} \right)dr = u_{{\text{A}}} \left( r \right)^{2} \cdot 2\uppi rdr . $$

Equation 3 indicates that the total intensity of the Gaussian beam is preserved when it is transformed to an annular beam^40^. The transformation function from an incident beam to an annular beam *u*_A_(*r*) is derived as

4 $$ u_{{\text{A}}} \left( r \right) = \sqrt {\frac{R - r}{r}} u_{{\text{G}}} \left( {R - r} \right) , $$

where *R* and *r* are the external and inner radii of the annular beam, respectively.

**Figure 2:**
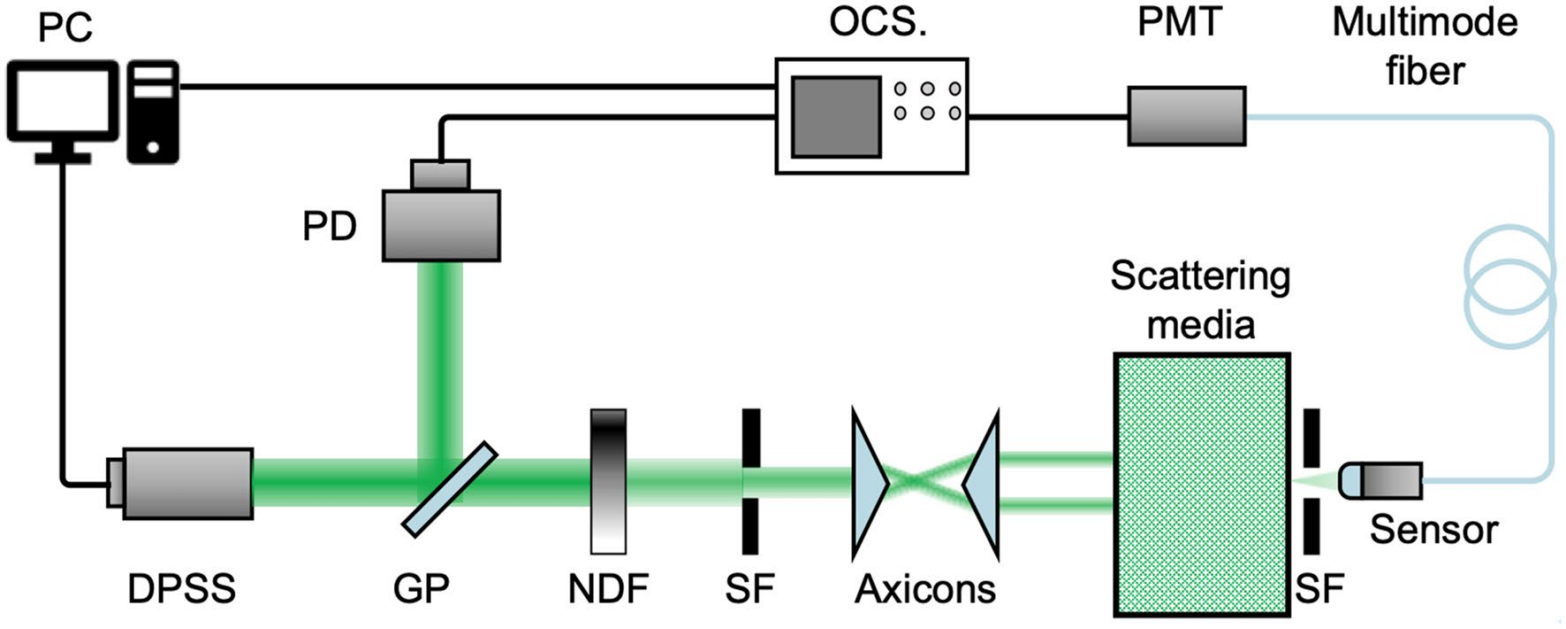
Experimental setup for the annular beam propagation in scattering media. A glass plate (GP) placed in front of the DPSS laser reflects part of the light toward a photodiode (PD), from which the trigger signal is obtained. A neutral-density filter (NDF) and a spatial filter (SF) are placed before the Axicons. The pair of Axicon prisms transforms the Gaussian beam to an annular beam.

Figure 2 shows the schematic diagram of the experimental system for the propagation of an annular beam in scattering media. Table 1 shows the specification of the optical elements in this system. A diode-pumped solid state (DPSS) pulse laser is utilized to generate a Gaussian beam. A neutral density filter is used to adjust the optical intensity of the beam. Axicon prisms with a large apex angle β = 150° are used to maintain the incident beam’s polarization and intensity distribution and to generate annular beams with diameters ranging from 8 to 45 mm. In this study, processed milk (milk fat 1.8% and casein 2.4%) is diluted with pure water to produce a dense scattering media with 0.1–30% concentrations. The milk fat and casein with an average particle size of around 1.1 μm and 0.1 μm are the main scatterers in this scattering media. The media’s scattering property depends on the milk fat since the casein is negligibly much smaller than the milk fat. The absorption is disregarded due to the small absorption coefficient of 0.0066 cm^−1^ at 40% media concentration^41^. Different media concentration and optical cells of lengths 1, 3, 5, 10, 20, and 30 cm are used. A receiver optics detects intensity distributions of the propagated light in scattering media just after the optical cell. A collimating lens of 6 mm aperture and a multiple-mode optical fiber with a core diameter of 50 μm are used as the receiver unit. The viewing angle of this receiver is narrowed to 5.5 mrad. Since the incident light is scattered in every direction in the scattering media, only the forward scattering light is detected. The intensity distributions of the propagated beam along the line perpendicular to the optical axis are measured. A photomultiplier tube (PMT R-636, Hamamatsu) and a high-speed sampling oscilloscope (DCA-J 86100C, Agilent) are used to detect and record the intensity distribution of the scattered light.

**Table 1:** Specification of the experiment system.

Light source	Annular beam converter	Receiver
DPSS laser CryLas, 1A532-1	Axicon prims: Apex angle 150° (± 10’)	Multiple mode optical fiber: Core diameter 50 μm
Wavelength 532 nm	Core diameter 51 mm	Collimating lens: Lens aperture 6 mm
Peak power 4.6 kW	Annular beam: Diameter range 8 to 45 mm	View angle 5.5 mrad
Pulse width 2 ns		
Repetition rate 10 kHz		

**Figure 3:**
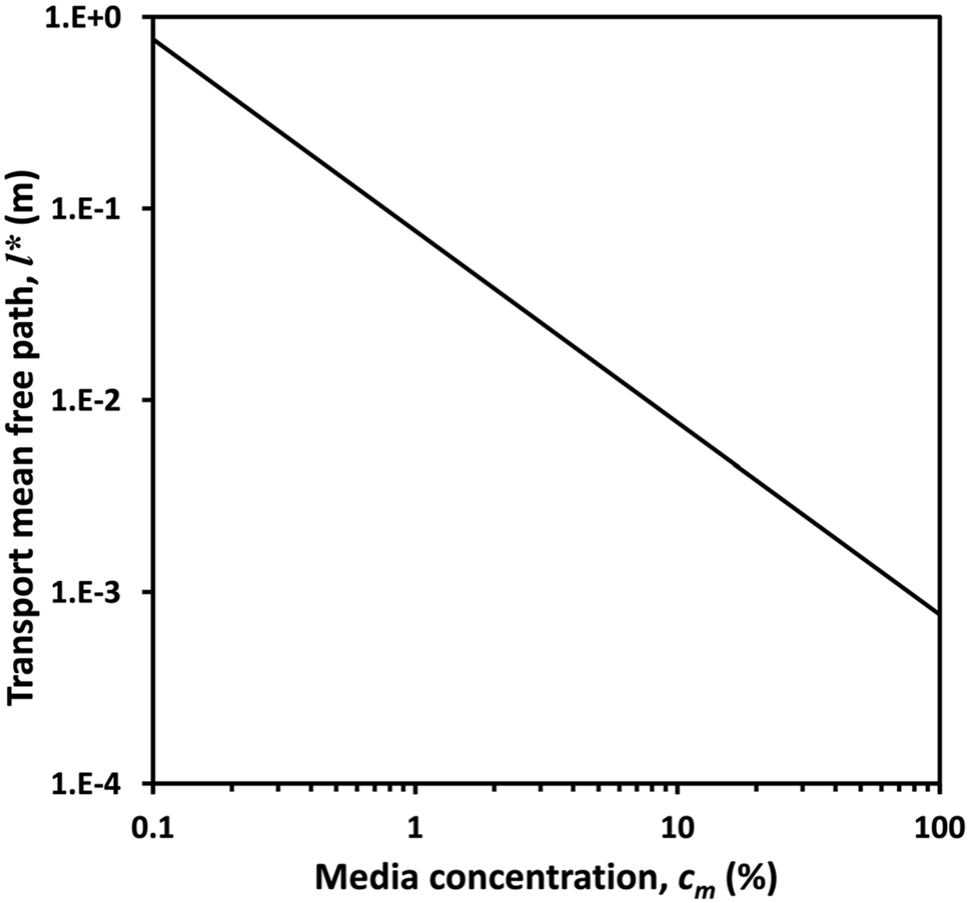
The variation between transport mean free path and media concentration from 0.1 to 100%.

Rayleigh and Mie scattering theory^42,43^ described the scattering property of a single small particle. In reality, the scattering effect is limited to single and multiple scatterings, especially in dense scattering media. TMFP is defined as the average distance the light travels to the next collision. This parameter, therefore, can be used to describe multiple scattering^33^. TMFP, l^*^, is quantified as 1/*μ*_s_ (1-g), in which *μ*_s_ is the scattering coefficient of fat in this scattering media. The parameter, *g* is the anisotropy factor that defines the degree of forward scattering and is expressed as

5 $$ {\text{g}} = \mathop \smallint \limits_{0}^{\pi } P\left( \theta \right)\cos \theta \cdot 2\uppi \sin \theta d\theta , $$

where *P*(*θ*) is the phase function at the scattering angle *θ*. TMFP, l^*^, can then be defined with the scattering coefficient as

6 $$ l^{*} = \frac{1}{{\mu_{s} \left( {1 - {\text{g}}} \right)}} = \frac{1}{{\uppi a^{2} QN\left( a \right)\left( {1 - {\text{g}}} \right)}} , $$

where *N*(*a*) is the number of particles with radius of *a* in a unit volume, *Q* is the scattering efficiency in Mie or Rayleigh Theory. The number of particles *N*(*a*) in the unit of volume is expressed as

7 $$ N\left( a \right) = \frac{{\uprho _{{\text{m}}} c_{{\text{m}}} c_{{\text{f}}} }}{{\frac{4}{3}\pi a^{2}\uprho _{f} }} , $$

where *c*_*f*_=1.8% is the percentage of fat in milk; *ρ*_*m*_=1.030 and *ρ*_*f*_ = 1.035 are specific gravities of milk and fat, respectively. Here, given the parameters of the scattering media, the number of particles per unit volume is *N*(*a*) = 2.57 × 10^16^ cm, where *c*_*m*_ is media concentration which can be changed from 0 to 100%. Figure 3 shows the variation between TMFP and media concentration obtained from Eq. 6 as l^*^ = 5.304 × 10/*c*_*m*_. The linear relationship resulted when logarithmic representations of media concentration and TMFP are plotted. The optical property of this scattering media can be discussed by TMFP value derived from the media concentration. For instance, to realize less than 10 mm TMFP, the media concentration must be at least 5% in our scattering media. In previous work^32^, the non-diffractive beam is maximized up to 2.4 mm TMFP, which is near to TMFP value of the human muscle^33^.

**Figure 4:**
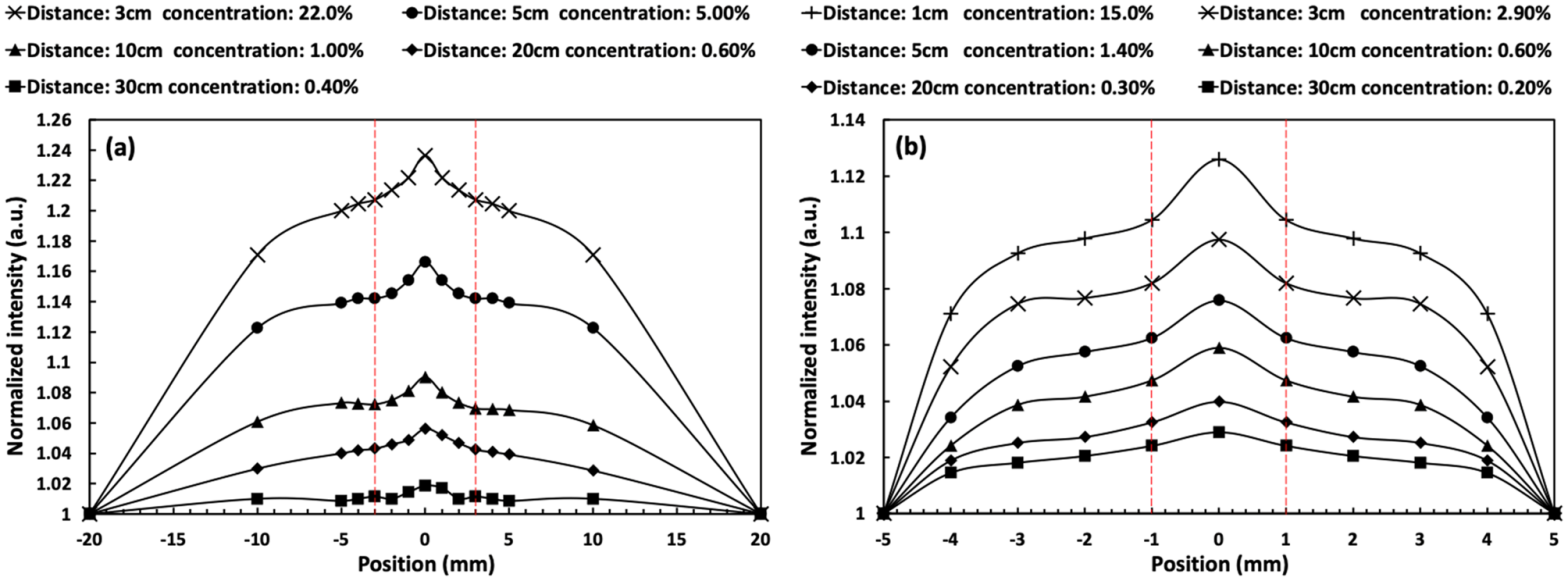
Observed transverse intensity distributions when the annular beam with (**a**) 40 mm and (**b**) 10 mm diameters are propagated in media cells with different lengths from 1 to 30 cm. The red dash-dotted lines indicate the regions (**a**) ± 3 mm and (**b**) ± 1 mm that are used to distinguish the central peak from the hill-like broaden signal. Here, position means the distance from optical axis.

Figure 4 shows the transverse intensity distribution of the forward scattered light under the annular beam with diameters of (a) 40 mm and (b) 10 mm. These intensity distributions are normalized at (a) 20 mm and (b) 5 mm from the optical axis, corresponding to the size of the incident annular beam. The main narrow central intensity and the hill-like broadened light intensity under the central intensity are constructed by forward and multiple scattered lights, respectively. This central peak is not an Arago spot because the width and intensity are kept during its propagation after the optical cell^32^. As explained below, these central peaks are NDBs resulting from the constructive interference of beams scattering in the forward direction^29,30^. Figure 4a shows the maximum NDBs generated at media concentrations of 0.4, 0.6, 1.0, 5.0 and 22% with their corresponding propagation distances of 30, 20, 10, 5, and 3 cm in scattering media, respectively. Here, NDB appeared at the center (maximum value at 0 mm position) with a width of around 6 mm. However, NDB could not be generated in a 1 cm cell under the propagation of an annular beam of 40 mm diameter. Numerical simulations of the propagation property of annular beams with different diameters in free space show that the maximum NDB is generated in a shorter distance when the diameter of an annular beam is smaller^44^. Also, in the case of scattering media with a constant propagation distance, it is anticipated that NDB can be generated from the propagation of annular beams with smaller diameters if the media concentration is low. To demonstrate this simulation result in the experiment, the diameter of the incident annular beam is changed from 40 to 10 mm in the propagation experiment. Figure 4b shows the transverse intensity distributions observed using optical cells of length from 30 to 1 cm. Maximum NDBs appeared when the media concentration is varied from 0.2 to 15% at each propagation distance. In the case of the 10 mm beam diameter, narrow peaks of 2 mm width are observed. Compared to the propagation property of an annular beam in free space, propagation of an annular beam in scattering media has the same characteristics, i.e., by using an annular beam with smaller diameter, the maximum central intensity of NDB is generated at shorter distance in scattering media.

**Figure 5:**
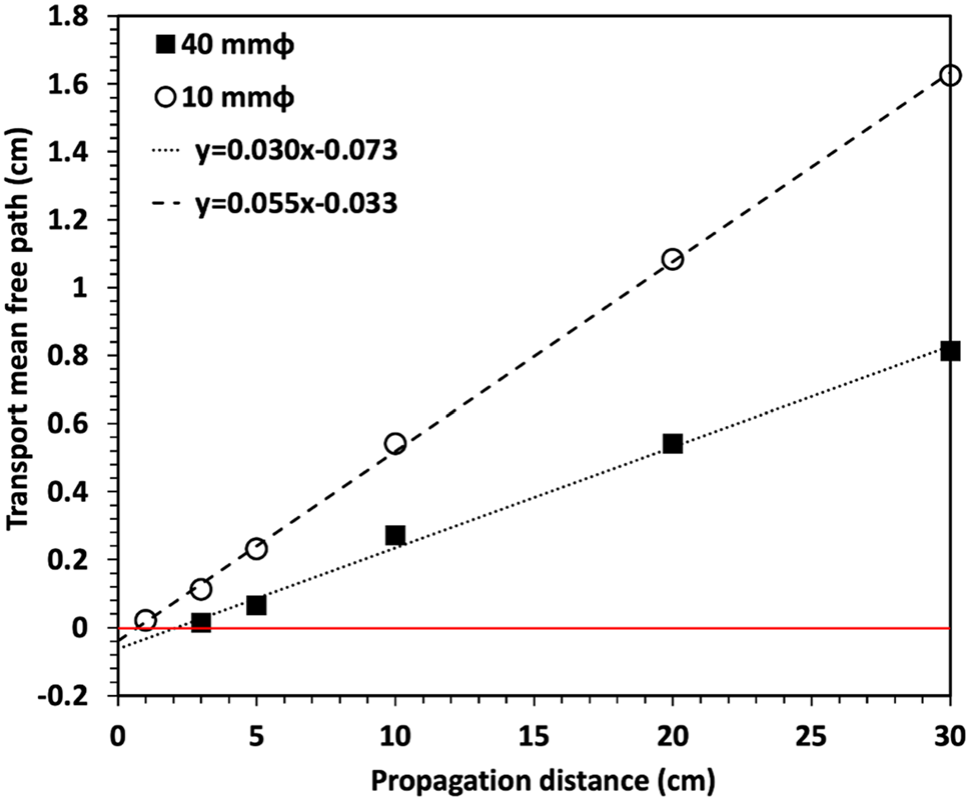
Relationship between the transport mean free path and relative propagation distance obtained from annular beams of 40 mm (squares) and 10 mm (open circles) diameter.

To analyze the relationship between TMFP and propagation distance, TMFP is derived using Eq. 6 for different media concentrations which generated the maximum NDB. Figure 5 shows the experimental results of TMFP with propagation distance obtained by using an annular beam of 40 mm and 10 mm diameters. Black squares show the experimental results of TMFP calculated by appropriate media concentration when an annular beam of 40 mm diameter is propagated in cells with lengths of 3 to 30 cm. A linear relationship *y* = *Ax* + *B* between the propagation distance and TMFP is obtained with a slope of *A* = 0.49 and a y-intercept of *B* = −1.23. The fitting coefficients indicate that since the TMFP is less than zero when the propagation distance is less than 2.5 cm, indicating that the scattering of annular beam cannot produce constructive interference to generate NDB. The open circles from the experimental data in 1 to 30 cm cells also show a linear property with its slope of *A* = 0.91 and y-intercept of *B* = −0.56 for an annular beam of 10 mm diameter. These linear relationships can be used to estimate the equivalent media concentration to generate the maximum NDB at different propagation distances. The line’s slope indicates that the optical depth, which can be related to the number of scattering events, is the same for any propagation distance if it has zero y-intercepts. However, if the y-intercept is nonzero, as shown in Fig. 5, the optical depth increases as the propagation distance decreases. This indicates that the y-intercept controls the minimum propagation distance to generate NDB. Different media will have different optical properties (e.g., particle size, index of refraction, etc.) and therefore, will produce different slopes and y-intercepts.

**Figure 6:**
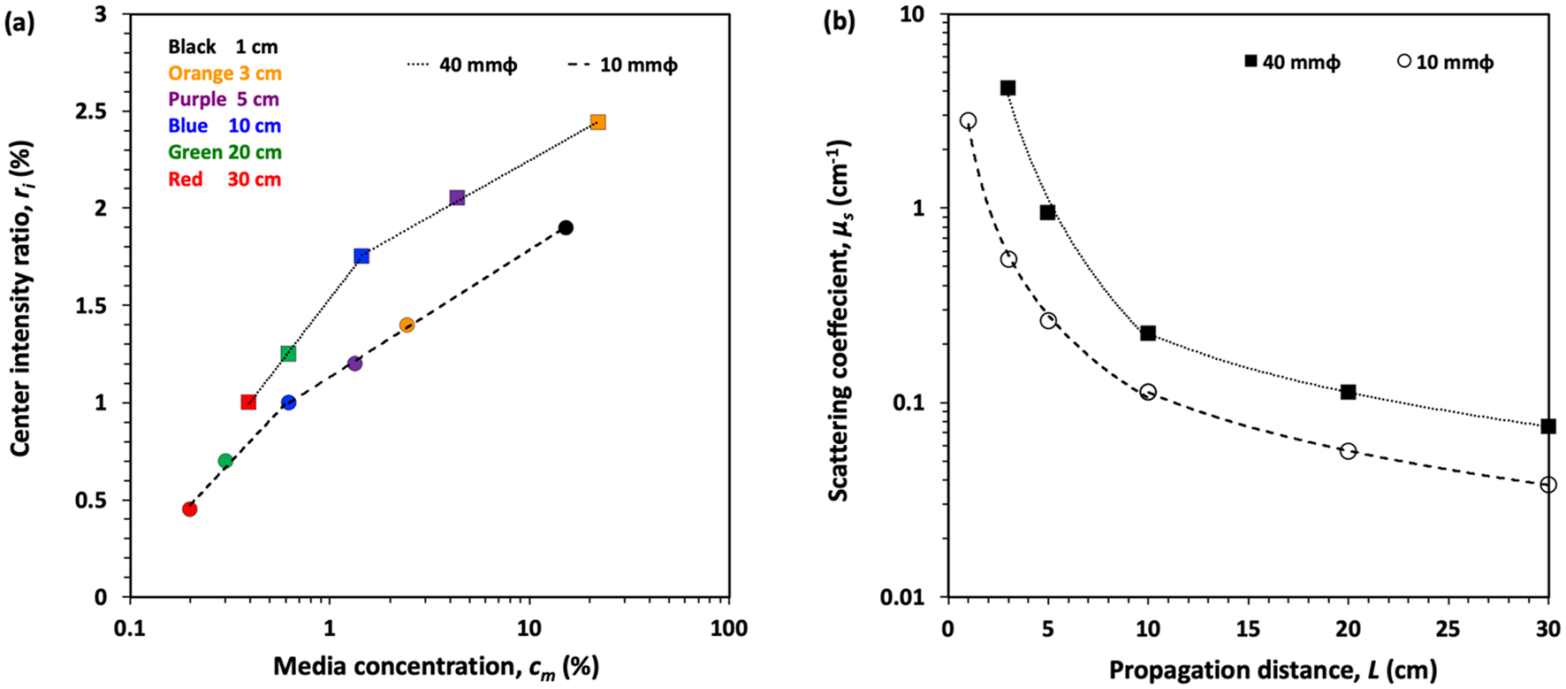
(**a**) Variation of the maximum center intensity ratio (*r*_*i*_) with the media concentration, and (**b**) the changes of scattering coefficient with the propagation distance observed from the annular beams with diameters 40 mm (black squares) and 10 mm (open circles) at different cell lengths (1–30 cm).

For evaluating the intensity of the non-diffractive beam, we define the center intensity ratio, *r*_*i*_, as^32^

8 $$ r_{i} = \left( {\frac{{V_{p} }}{{V_{s} }} - 1} \right) \times 100\% , $$

where *V*_*p*_ and *V*_*s*_ are the normalized intensity at the position of peak and trough of the beam intensity profile. This ratio can also mean as the intensity ratio between the non-diffractive beam and the multiple scattering lights at the center position. Figure 6a shows the variation of the maximum center intensity ratio, *r*_*i*_ with different media concentrations when annular beams with diameters 40 mm and 10 mm are propagated in 1 to 30 cm and 3 to 30 cm optical cells, respectively. The curve in Fig. 6a can be defined by the logarithmic equation (Eq. 9)

9 $$ r_{i} = A^{{\prime }} \log \left( {c_{m} } \right) + B^{{\prime }} , $$

where *A'* and *B'* are the coefficients that result after performing the logarithmic fitting. The slopes of the curve for cell lengths less than 10 cm are the equal (*A'* = 0.5) for both beam diameters but the y-intercept values are not. This same case holds for cell lengths greater than 10 cm (*A'* = 0.27). Since multiple scattering contributes greater in higher media concentration, slope of the center intensity ratio is lower for cell lengths greater than 10 cm. This implies that beams with different diameters have the same propagation behavior when they are propagating in the same media. The change of the slope from 0.50 to 0.27 can be presumably explained by the significant increase in multiple scattering for higher concentrations. Figure 6b shows the variation of scattering coefficient with propagation distance. The scattering coefficient is defined as the reciprocal of TMFP, l^*^ = 1/*μ*_s_ (1-g). The nonlinear curves indicate that for shorter propagation distances (i.e., < 10 cm), the scattering coefficient is significantly higher, indicating the occurrence of multiple scattering effects due to higher optical depths. For both curves, the increase of scattering coefficients with propagation distance is the same (i.e., the same slopes).

**Figure 7:**
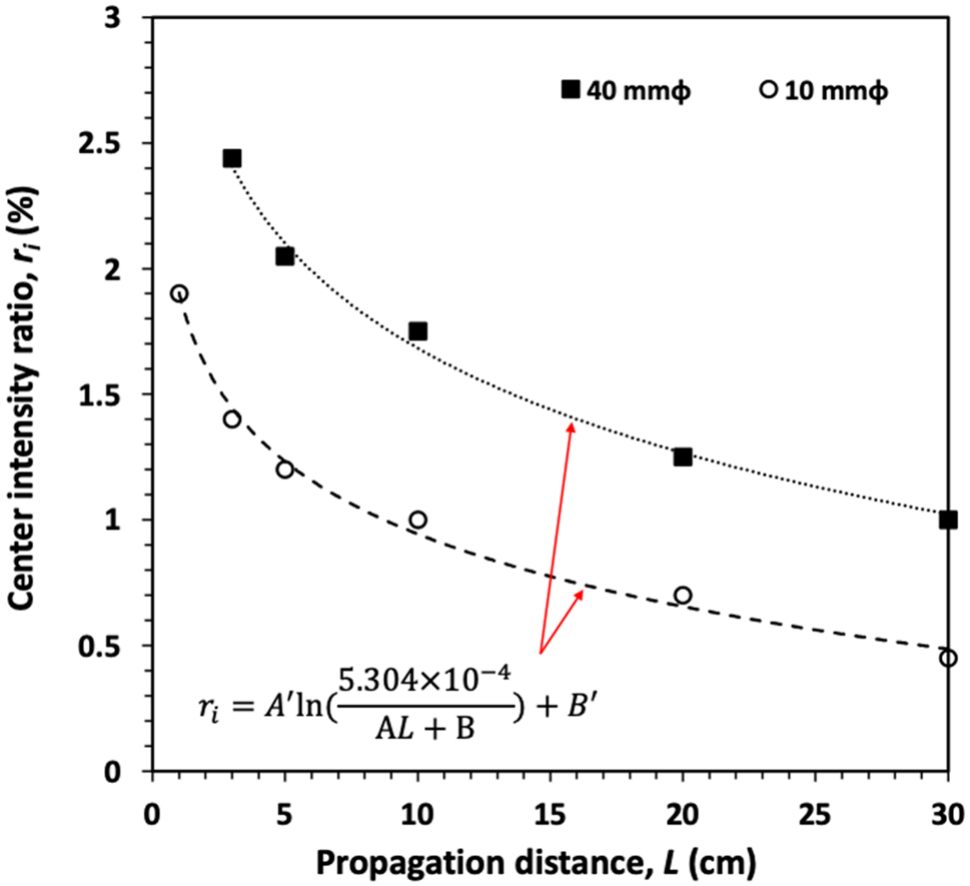
(**a**) Variation of the maximum center intensity ratio (*r*_*i*_) and relative propagation distance observed from the annular beam with diameters 40 mm (black squares) and 10 mm (open circles) in different cell lengths (1–30 cm).

Figure 7 shows the variation of the maximum center intensity ratio with propagation distance for annular beams with diameters of 40 mm (black squares) and 10 mm (open circles). The media concentration can be converted to propagation distance to produce the curves in Fig. 7. The equation in Fig. 7 is the same as Eq. 9 but is expressed in terms of the linear fit between the transport mean free path and propagation distance in Fig. 5. These two curves have the same slope with the propagation distance. This is expected since the beams are propagating in the same media. The curve representing the 40 mm beam has higher values of center intensity ratio for a fixed propagation distance. This can be attributed to the size of the beam. Beams with larger diameters undergo higher scattering events. The equation representing the curves for different diameters can be used to deduce the central intensity of adequate NDB at different propagation distances.

## Conclusion

An experimental approach to realize the macroscopic sensing in scattering media is reported in this paper. We have experimentally explored the relationship among the generation characteristics of adequate NDB at different diameters, media concentrations and propagation distances. Here, the “adequate NDB” is defined as the maximum central intensity under a certain propagation distance in scattering media. The generation condition of adequate NDBs is experimentally indicated by the linear relationship between propagation distance and transport mean free path, and also the relationship between center intensity ratio of NDB and propagation distance. These relationships have common trends even if the diameter of the annular beams is changed. The adequate NDB can be generated by a small diameter (10 mm) annular beam when the media concentration is less than 15%. From the experiment, the relationship between TMFP and propagation distance in scattering media can be explored. This provides information on the generation conditions of adequate NDB at different propagation distances. The change of center intensity ratio with media concentration is the same for different beam diameters. A larger beam diameter has a higher center intensity ratio because it has a larger number of scattering events. The relationship between the center intensity ratio and propagation distance for different beam diameters can be modeled using an equation derived from experimental results. This equation can be used to infer the central intensity of the adequate NDB at different propagation distances.
